# Modeling of trees failure under windstorm in harvested Hyrcanian forests using machine learning techniques

**DOI:** 10.1038/s41598-020-80426-7

**Published:** 2021-01-13

**Authors:** Ali Jahani, Maryam Saffariha

**Affiliations:** 1 Research Center of Environment and Sustainable Development, College of Environment, Tehran, Iran; 2 Department of Rangeland Management, College of Natural Resources, University of Tehran, Tehran, Iran

**Keywords:** Forest ecology, Forestry, Ecological modelling, Forest ecology, Forestry

## Abstract

In managed forests, windstorm disturbances reduce the yield of timber by imposing the costs of unscheduled clear-cutting or thinning operations**.** Hyrcanian forests are affected by permanent winds, with more than 100 km/h which cause damage forest trees and in result of the tree harvesting and gap creation in forest stands, many trees failure accidents happen annually. Using machine learning approaches, we aimed to compare the multi-layer perceptron (MLP) neural network, radial basis function neural network (RBFNN) and support vector machine (SVM) models for identifying susceptible trees in windstorm disturbances. Therefore, we recorded 15 variables in 600 sample plots which are divided into two categories: 1. Stand variables and 2.Tree variables. We developed the tree failure model (TFM) by artificial intelligence techniques such as MLP, RBFNN, and SVM. The MLP model represents the highest accuracy of target trees classification in training (100%), test (93.3%) and all data sets (97.7%). The values of the mean of trees height, tree crown diameter, target tree height are prioritized respectively as the most significant inputs which influence tree susceptibility in windstorm disturbances. The results of MLP modeling defined TFM_mlp_ as a comparative impact assessment model in susceptible tree identification in Hyrcanian forests where the tree failure is in result of the susceptibility of remained trees after wood harvesting. The TFM_mlp_ is applicable in Hyrcanian forest management planning for wood harvesting to decrease the rate of tree failure after wood harvesting and a tree cutting plan could be modified based on designed environmental decision support system tool to reduce the risk of trees failure in wind circulations.

## Introduction

Windstorm disturbances occur when sudden changes happen in wind loading and trees have not acclimatized to new conditions of the site^1^ in thinned or adjacent to clear-cut stands. In managed forests, windstorms reduce the yield of timber by imposing the costs of unscheduled clear-cutting or thinning operations^2^. Windstorms cause significant changes in forest management plans, climate change^3^ and carbon storage of forests^4^ especially in temperate regions^5^. Therefore, we need to model the response of trees to wind pressure to achieve reliable prediction and develop new strategies in forest management^6^. We believe that it is possible to develop mathematical models to predict the role of wind in trees stability by discovering the response of trees to sudden windstorms. This approach allows forest managers to predict the impact of forest activities or silvicultural plans on tree stability in sudden windstorms. The modeling approach quantitatively describes the relationship between tree attributes and tree susceptibility under windstorm tension. Recently, mechanistic models have been developed for forest ecosystems where the natural phenomena are influencing forest succession^7,8^. These models are mainly based on stand and tree characteristics, and aim to identify trees having potential for failure or uprooting^1,9^.

The main advantages of artificial neural networks (ANNs) in comparison with classic logistic regression are summarized in data analyzing which results in developing more accurate predictive models^10^. ANNs model the structure of the human brain in data analysis and create a neural structure as the human brain analyses data in parallel by several neurons. ANN is structured by a series of mathematical equations to simulate environmental processes such as wind-susceptible tree identification. ANNs and machine learning techniques are some algorithms that learn from samples and data without relying on rules-based system programming; while common statistical modeling formulates the relationships between input and output variables in the form of mathematical equations*.* ANNs have a variety of advantages, such as the ability to implicitly distinguish complicated nonlinear relationships between inputs (independent variables) and outputs (dependent variables). ANNs also use multiple training algorithms and rules and less formal statistical training. These methods have very distinctive potential to determine all possible interactions between tree failure and ecological variables^11^. Considering that, ANN methods resulted in a significant increase in prediction accuracy (approximately up to 80% of water discharge studies) in comparison with multiple regressions^12^.

The literature review reveals some advanced technologies which can predict the relationship between human activities and ecological processes. For example, Jahani et al.^13^ determined MLP, RBF and SVM, as the most accurate models in the ecological process modeling to predict vegetation density loss in response to the tourist activities, based on the ecological conditions of national parks. Considering the lack of valid long term data sets (noisy data) and a considerable number of variables, which influence tree failure, machine learning techniques and artificial intelligence will be applicable in susceptibility assessment and modeling in many ecological phenomena^14–16^. For example, Jahani^11^ developed SFHCM (Sycamore Failure Hazard Classification Model) model for hazardous tree classification in urban green spaces using an artificial neural network technique. SFHCM classifies the susceptibility of Sycamore trees under wind pressure in four classes of tree failure within a year containing 1. Offshoots, 2. Branches, 3. Tree crown and 4. Whole tree failure. However, machine learning techniques, such as Multi-Layer Perceptron (MLP), Radial Basis Function Neural Network (RBFNN) and Support Vector Machine (SVM) has been applied in many types of ecological researches (e.g. Jahani and Saffariha^17^; Hong et al.^18^). We aimed to compare the MLP, RBFNN and SVM models for prediction of tree failure in forest ecosystems. The main objectives were to: (1) model tree failure potential in the windstorm of forest lands; (2) compare different machine learning techniques to identify the most accurate model; (3) prioritize the model inputs (tree and stand variables) using sensitivity analysis of the model; and (4) designing environmental decision support tool for wind-susceptible tree identification.

## Materials and methods

### Site selection

**Figure 1:**
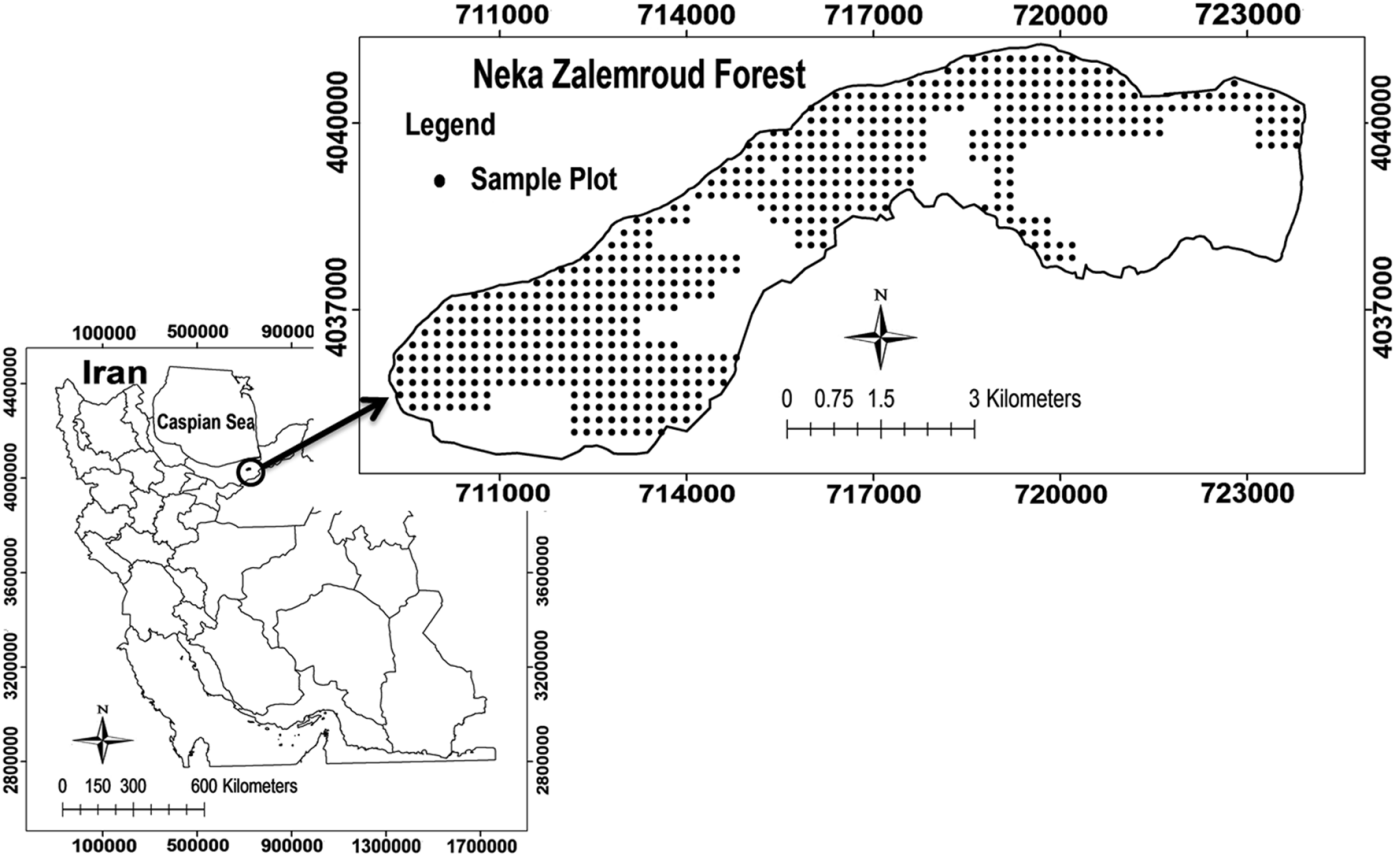
The location of study area and sample plots (QGIS 3.12.0, https://www.qgis.org/en/site/).

Hyrcanian temperate forests, dominated by old broadleaf trees, are located in the north of Iran adjacent to the Caspian Sea. We selected the Neka Zalemroud forest in Mazandaran province as the study area for this research (36° 26′ 09″ to 36° 30′ 47″ N latitude and 53° 20′ 34″ to 53° 31′ 51″ E longitude) as the boundaries of sampling area have been illustrated in Fig. 1 by the authors. This forest has been covered with 2533 hectares of old broadleaf trees such as *Fagus orientalis, Carpinus betulus, Quercus castanafolia, Acer velutinum, Acer cappadocicum, Parrotia persica* and some other species. This region is affected by permanent winds and the maximum wind speed is in the range of 10 to > 30 m/s. The windstorms with more than 100 km/h cause damage forest trees, including uprooting or stem breakage. The windstorms cause many tree failures annually, which is in result of the tree harvesting and gap creation in forest stands. Hence, we aimed to identify the uprooted or stem broken trees using the sample plots data after the windstorm with 100 km/h (4 h) on 21 March 2018.

### Methods

Data from field measurement of 600 sample plots in the study area were summarized before and after the windstorm in March 2018. ARC MAP 9.3 software was used to spatially locate the sample plots on the forest map. Also, the slope of plots was defined by topography map (1:50,000) in this software. The permanent sample plots were created by Department of Natural Resources of Mazandaran Province (in the structure of the forest management plan) as a part of government-sponsored “permanent sample plot” protocol to monitor the changes in the number of trees and species^48^. In this protocol, the centers of these sample plots are marked with a metal rod and the geographical coordinates are recorded. The attributes of all trees in the sample plots are recorded consisting of species, diameter at the breast height, tree's height, number of trees, distance to the center of the plot, crown diameter and land, soil and climate characteristics of the plot location. On average, in each plot, there are about 10 to 20 trees with a diameter of more than 7 cm (marketable size), and using the recorded data of the trees, it is possible to identify each tree in subsequent monitoring. As the protocol dictates, preliminary data were recorded on the stage of creating plots by experts from the Provincial Department of Natural Resources in 2016^49^. After the windstorm on 21 March 2018, these plots were investigated by the authors to find damaged and undamaged trees. The area of each sample plot was 1000 m^2^ and in the shape of the circle with 17.84 m radius. These plots were investigated to record harmed trees which were uprooted or stem broken. Sample plots are clustered into susceptible (with harmed trees) and unsusceptible (without harmed trees) plots. We recorded the forest stand attributes, as well as tree attributes, which influence the likelihood of trees to be damaged in windstorms. Therefore, we investigate the damaged and resistant trees in the plots within 1000 m^2^ area. The stand variables were recorded in the plot area such as the mean of the trees' height in the plot. Some plots (306 plots) were determined where the damaged trees were observed and some others (294 plots) were determined where the target tree in the middle of the plot was undisturbed. In a literature review, we found that stand and tree characteristics influence wind-susceptibility of trees (such as tree diameter and height, spread crown area, rooting system, soil type, stand density, topography, etc.^1,3,19–21^). Therefore, 15 variables in 600 sample plots were recorded that are divided into two categories: 1. Forest stand variables: Plot Slope (PS) (%), Soil Depth (SD) in plot area (cm), trees Mean Diameter at the Breast Height (MDBH) at plot (cm), trees Mean Height (MH) in plot (m), trees Density (De) in plot (Number of trees), trees Diversity (Di) in plot (Number of tree species), Number of Thicker trees in diameter (than the target tree) (NTh), and Number of Taller trees (than the target tree) (NTa).

2.Tree variables (bigger than marketable size): Tree Area (TA) (occupied area by the tree) (m^2^), Tree Diameter at the Breast Height (TDBH) (cm), Tree Height (TH) (m), Tree Crown Diameter (TCD) (m), Mean Distance from Neighbor trees (MeDN) (m), Minimum Distance from Neighbor trees (MiDN) (m), and Maximum Distance from Neighbor trees (MaDN) (m). Tree heights and some more data were recorded in permanent sample plots before the windstorm. Therefore, we used the recorded data at the stage of creating plots, for damaged trees. In fact, 10 to 20 trees are recorded in each plot and using the recorded data of the trees (species, diameter, distance to the center of the plot, etc.) it is possible to identify target trees (damaged or undamaged) in subsequent monitoring. There are some other factors that influence wind-susceptibility of trees such as forest edge, tree diversity, and land form. In this research, we were looking for the impact of forest plan activities on tree failure; therefore, we neutralized forest edge effects by selecting sample plots inside the forest stands which are far from the forest edges. In land form variables, land slope was considered in stand variables, but altitude and geographical aspect of the hill, as well as tree diversity, were omitted because of limited variation in the samples.

Tree Failure Model (TFM) was developed by recording 15 variables of the trees and forest stands in 600 selected sample plots. In fact, we designed three TFMs with three modeling techniques to achieve the most accurate one based on model accuracy assessment. The damaged trees in the plots were identified by two features: (1) The tree was uprooted by wind forces along the windstorm. (2) The tree was uprooted or stem broken. Leaning trees (under windstorm force) were also counted as uprooted trees. The damaged tree was chosen as the target tree in susceptible plots (plots with a damaged tree). Also, the central tree was selected as the target tree in the wind-unsusceptible plots (plots without damaged tree). Indeed, the output of TFM will be in two classes of damaged trees (1) and stable trees (0). Hence, the response or output of the model will be a discrete class {0,1}. The accuracy of the model is assessed by confusion matrices which detect the number of accurate and false classifications of sample trees.

The new mathematical modeling approaches and machine learning techniques are needed to cover limitation in data collection and forest inventories. Indeed, ANNs are over 50 years old, but just not often applied yet. History of ANN development represents this fact that learning algorithms and structure of neural networks are developed every year. Therefore, we developed artificial intelligence techniques in natural phenomena modeling^11,17^ namely MLP, RBFNN, and SVM.

Machine learning techniques rely on a specific concept that is "a set of weak learners develop a single strong learner" (by Freund and Schapire^22^, Breiman^23^ and Breiman et al.^24^). As it is known, a weak learner is a classifier correlating slightly with the real (target) classification; while a strong learner is well correlated with real (target) classification. Machine learning algorithms are trying to combine weak classification rules in a one strong classification rule. Based on this approach, we used 15 variables (even if we believe that these variables are not related to tree susceptibility in the wind) and tested some algorithms and variable weights to make weak learners or rules and combining them into one strong rule in the structure of three machine learning techniques.

### Multi-layer perceptron (MLP) neural network

ANNs use different methods, such as feed-forward, backward, recurrent and other, to teach the network for output prediction. MLP is a multi-layer form of Feed-forward neural networks without any cycle or loop. In Feed-forward neural networks, the information analysis is performed in one direction from the input layer, through the hidden layer to the output layer^47^. In this learning method, the errors of the network propagate from the output layer to inputs to revise the weights of input variables. MLP is a multi-layer Artificial Neural Network (ANN) model with self-learning mechanism which uses samples for classification. Indeed, MLP has been using some interconnected processing elements that are called PEs (Processing Elements). MLP learns by using samples and transfer functions which are applied between neurons and hidden layers in a computer program^17^. In the training process, each PE receives signals periodically from other PEs and sends the new signal to other processors. Considering inputs, MLP adjusts the weights of neurons continuously, and the learning process is completed.

We used some activation functions (such as logarithmic sigmoid, hyperbolic tangent, and linear transfer functions) which determine the relation between inputs and outputs and these functions were tested to achieve the best performance of MLP. Back Propagation (BP) method propagates the error of outputs to the input layer where the first random weights have been assigned. The weights of the network inputs will be justified until the best performance of the network is reached; and after that, the learning process will be completed^7,11^. Errors between Ynet (MLP output) and Y (real class of tree failure) are decreased by BP when the weight of neurons or Processing Elements (PEs) (w) and input variables (x) come to the best performance, and the output of jth PE on the kth layer (PEkj) will be achieved by Eq. (1):

1 $$net_{j}^{k} = \mathop \sum \limits_{i = 0}^{n} w_{ji} x_{ji}$$

Transfer functions are used in the structure of network, and neuron output value is determined by (Eq. 2).

2 $$Y_{net} = \smallint net_{j}$$

Finally, weights of t samples will be adjusted by delta rule which has been summarized in Eq. (3).

3 $$w_{ji}^{t} = w_{ji}^{t - 1} + \Delta w_{ji}^{t}$$

By using the ANN function in MATLAB R2013b, 360 uniformly distributed random samples (60% of 600 samples) were defined as training data set. 120 evenly distributed random samples (20% of 600 samples) were defined as validation data set, and 120 samples (20% of 600 samples) were determined as test data set. All data were normalized to the interval of 0 to 1 using the Min–Max technique by mapminmax function in MATLAB R2013b (Refer to Demuth and Beale^25^ for MATLAB codes for MLP neural network development and related preprocessing algorithms).

### Radial basis function neural network (RBFNN)

Radial basis function neural network is architecturally similar to the MLP with different activation function in the hidden layer. RBFNNs have been used in function approximation and classification in researches of the last decade^13,26–28^. RBFNN uses samples in two data sets of training and test. The radial function is applied in each neuron of the hidden layer; and the number of neurons depends on input matrix of variables. Considering two classes of trees failure (0 and 1) in this research, we have two output layers in the structure of RBFNN. Gaussian function is the most frequently used function in the hidden layers of RBFNN^13,27^. The Gaussian function can find the center of circular classifiers successfully. The Gaussian function regulates the centre of mentioned circular classifiers by Eq. (4).

4 $$R_{j} \left( x \right) = exp\left( {\frac{{||x - a_{j} ||^{2} }}{{2\sigma^{2} }}} \right)$$

In Eq. (4), input variables are structured in the matrix "x", radial basis function has been defined as R_j_(x), centre of RBF function is presented as aj, and we have a positive real number as "ϭ". The outputs of network will be calculated by an output function Eq. (5).

5 $$y_{k} = \mathop \sum \limits_{j = 1}^{m} w_{jk} R_{j} \left( x \right) + b_{j}$$

In Eq. (5), the number of calculation nodes in the structure of hidden layers (j), the number of neurons (m), the weights of neurons (w_ik_), and a bias value (bj) have been used to calculate output (y_k_).

Neuron weights (wjk) are updated continuously to decrease output errors until network training process comes to end. Network performance is calculated when the number of neurons and the weights of neuron or layers are fixed^13^. (Refer to Demuth and Beale^25^ for MATLAB codes for RBF neural network development and related preprocessing algorithms).

### Support vector machine (SVM)

SVM is one of the machine learning techniques that requires quite a lot of data for training, but this method also provides more accurate results than other methods when the volume of training data is limited^29,30^. Therefore, SVM has been used for modeling in this paper to deal with this issue with the collected data along forest inventory.

As a classifier technique, SVM aims to determine the largest margin in decision boundaries that could separate classes of decision^31^. SVM is looking for the largest margin in the boundaries of classification when the uncertainties in the decision are expected^27^. This method of prediction minimizes the probability of over-fitting in classes limits of tree failure.

We have two datasets of training and test in the structure of SVM. The values of target are structured in a n-dimensional matrix so it is possible to find the most accurate boundaries and margins. Equation (6) is the SVM model and equation parameters define: y(x) = SVM output, α_i = a multiplier, K = kernel function, and b = threshold parameter.

6 $$y\left( x \right) = \mathop \sum \limits_{i = 1}^{n} \alpha_{i} K\left( {x_{i} ,x_{j} } \right) + b$$

Then we defined Gaussian Radial Basis Function (RBF) in Eq. (7), in the context of non-linear SVM. As we know, RBF is the most popular function in the context of SVM with remarkable ability to control generalization of SVM classifier.

7 $$K\left( {x_{i} ,x_{j} } \right) = {\text{exp}}\left( { - \gamma\| x_{i} - x_{j}\|^{2} } \right)$$

The parameters of Eq. (7) are: xi and xj = samples and γ = kernel parameter.

Finally, primal problem in Eq. (8) should be minimized to achieve the most accurate SVM for tree failure prediction.

8 $$\frac{1}{2}\|w\|^{2} + C\mathop \sum \limits_{i = 1}^{n} \xi_{i}$$

In Eq. (8), the parameters are: 1/2||w||2 = the margin, Σξi = training errors and C = the tuning parameter.

SVM uses samples in two data sets of training and test. The classes of the target will be summarized in a n-dimensional matrix to determine the nearest classification boundaries and margins. (Refer to Demuth and Beale^25^ for MATLAB codes for SVM neural network development and related preprocessing algorithms).

### Model selection

MLP, RBFNN, and SVM models were run on the training dataset with 15 tree variables as inputs and tree failure classes in 600 selected trees as output. To evaluate prediction accuracy of the MLP, RBFNN, and SVM models, we used the confusion matrix to determine the percentage of accuracy in target tree classification. Also, the number of trees with accurate and failed classification will be detected^11^.

### Sensitivity analysis

Sensitivity analysis was designed to prioritize the most accurate model variables with respect to the significance of variables in output. Sensitivity analysis defines the usefulness of variables in model predictions. In sensitivity analysis, we changed each variable in the range of standard deviation with 50 steps while the other variables were fixed at the value of the average. Then, the standard deviation of outputs for each variable changes was measured as model sensitivity for that variable. Variables with high value in the outputs standard deviation are the most important variables with more influence on model outputs. The trend of model output changes with changing the most significant variables, in the range of standard deviation (50 steps) was illustrated in some figures to find out the way that model outputs are changing with variable changes (negatively or positively) (Refer to Kalantary et al.^27^ and Jahani et al.^13^.

### Environmental decision support system (EDSS) tool

Finally a user friendly GUI (Graphical User Interface) tool was designed as an EDSS for susceptible tree identification in windstorms. It is applicable for forest managers who are looking for hazardous trees to plan for tree protection and increase the forest stands resistant against windstorms. ANN models use a huge matrix of weights; so model execution should be in the mathematical software (in this research MATLAB R2013b). Users, who are not familiar with the software, need a simple tool to run the model on new samples and get the results of prediction. To design EDSS tool, we developed a GUI extension in MATLAB R2013b software. With this tool, users enter the values of trees and stands variables (based on forest inventory data in other target forests) and the susceptible trees will be identified only by pushing a button. The model will be run on the data and the model outputs for each tree will be appeared in a table (0 or 1).

## Results

Totally 306 damaged trees (306 sample plots) were identified in this study. On the other hand, 294 plots did not contain the damaged tree; so the central tree in the plot was recorded as stable tree. The recorded variables of the tree and stand, which have been used as model variables, are illustrated in Table 1. Indeed, the minimum, mean and maximum of model variables define the limits of model validity in practice.

**Table 1:** Statistical data of tree and stand variables.

Stand variables	Mean ± Standard error	Min	Max	Tree variables	Mean ± Standard error	Min	Max
Plot slope (%)	10.79 ± 0.22	1	30	Tree Area (m2)	171.93 ± 2.18	61	295
Soil Depth (cm)	28.47 ± 0.44	10	50	Tree Diameter at the Breast Height (cm)	95.82 ± 0.84	35	142
Trees Mean Diameter at Breast Height (cm)	31.42 ± 0.26	15	50	Tree Height (m)	25.89 ± 0.07	19	32
Trees Mean Height (m)	19.3 ± 0.14	11	28	Tree Crown Diameter (m)	10.34 ± 0.08	5	16
Trees Density (N)	12.42 ± 0.11	5	19	Mean Distance from Neighbor Trees (m)	7.72 ± 0.05	4.5	9.9
Trees Diversity (N)	2.48 ± 0.03	1	4	Minimum Distance from Neighbor Trees (m)	4.44 ± 0.05	1.5	6.8
Number of Thicker Trees in diameter than the target tree (N)	1.63 ± 0.04	0	5	Maximum Distance from Neighbor Trees(m)	14.01 ± 0.04	11.5	15.8
Number of Taller Trees (N)	1.52 ± 0.03	0	5				

In this paper, the accuracy of three predictive models, namely MLP, RBFNN, and SVM, in tree susceptibility assessment and prediction, was tested. The most accurate model discovers tree and forest stand attributes which result in a greater chance of tree failure. Sensitivity analysis of the best model aims to find out which parameters and how influence the chance of tree failure in windstorm events.

### Prediction performance of MLP

The number of neurons and hidden layers, activation function, and training method may lead to different MLP prediction performances (Table 2). The best MLP structures, its' accuracies and training functions have been reported in Table 2. Considering the accuracy of classification in training and test data sets (Table 2), the most successful training function is SCG and the topology of MLP is (15-27-1) which means that 15 variables as inputs, 27 neurons in hidden layer, and one neuron (failure class) in output layer. The tangent hyperbolic transfer function was detected as the best estimation function in hidden and output layers.

**Table 2:** The results of parameters tuning in MLP, RBFNN and SVM structure.

Activation function	Training function	Structure	Test set	Training Data
			Accuracy (%)	Accuracy (%)
Tanh-Tanh	Scaled conjugate gradient (SCG)	15–27-1 MLP	93.3	100
Spread	Neurons	Structure	Test set	Training Data
495	35	RBFNN	90.6	94.8
C	γ	Structure	Test set	Training Data
200	24	SVM	93.4	97.9

**Figure 2:**
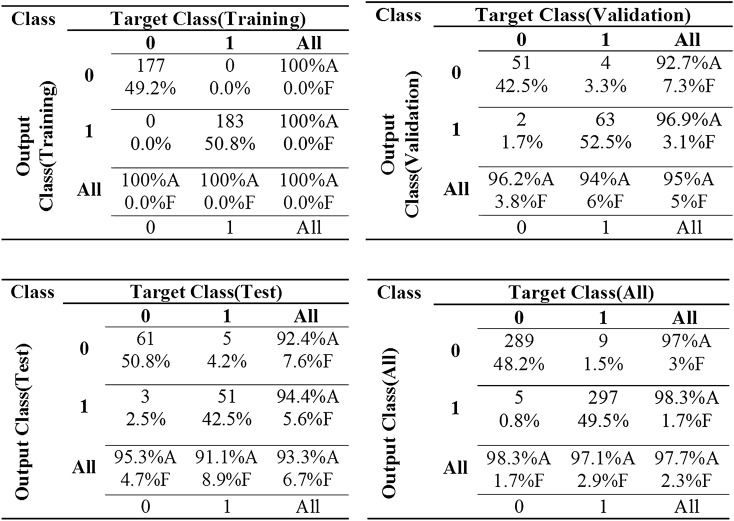
The confusion matrix of MLP neural network.

Considering the confusion matrix in Fig. 2, we find remarkable accuracy of classification in MLP model for three data sets of training, validation, and test. The designed model resulted in 100% successful in tree failure classification in the training data set. The accuracy of classification was 95% in the validation data set, 93.3% in the test data set, and 97.7% in the all data sets. MLP failed six samples of 120 samples in the validation data set, eight samples of 120 samples in the test data set, and 14 samples of 600 samples in the all data set.

### Prediction performance of RBFNN

RBFNN has been designed with a feed-forward structure, input layer, a single hidden layer with Gaussian transfer function and an output layer. RBF neural network minimizes the error of prediction by using a variety of parameters which include: the number of neurons, spreads of radial basis functions, and mean squared error goal. In this research, RBFNN parameters were defined to minimize the error of prediction: the number of neurons = 5–250, the spread of radial basis functions = 15–600 and Mean squared error goal = 0. The best results of RBFNN have been represented in Table 2.

**Figure 3:**
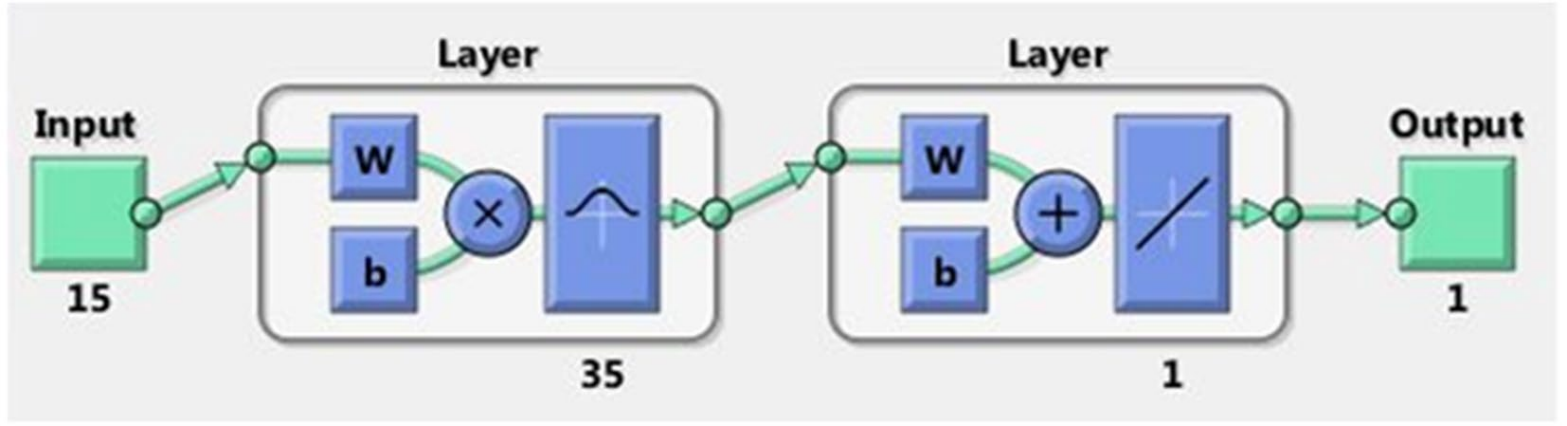
The best structure of RBFNN model (MATLAB R2013b, http://www.mathworks.com).

Considering the accuracy of classification in training and test data sets (Table 2), the most successful topology of RBFNN is presented in Fig. 3. The optimized RBFNN has been structured with 15 variables as inputs, 35 neurons in hidden layer with Gaussian transfer function, and one neuron (tree failure class) in output layer with a linear transfer function. The optimized RBFNN is illustrated at Fig. 3 in MATLAB R2013b software.

**Figure 4:**
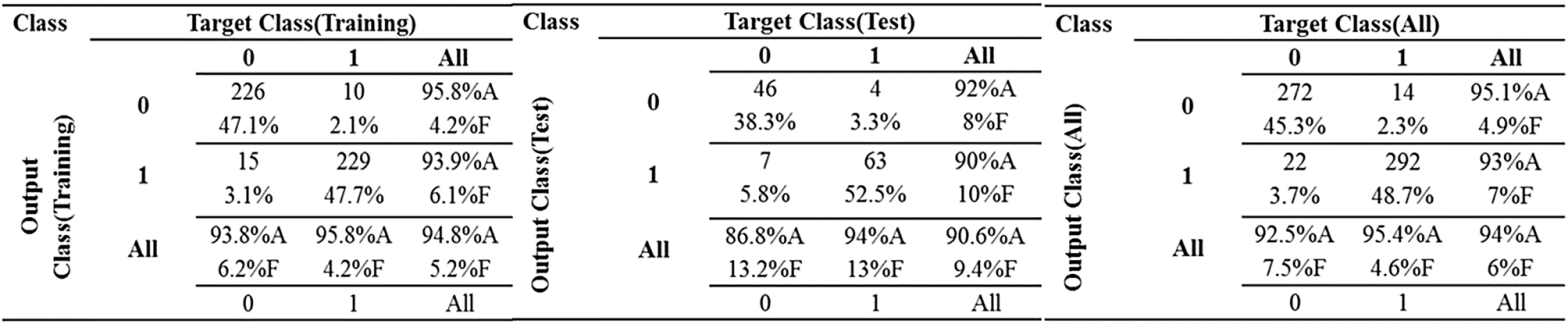
The confusion matrix of RBFNN neural network.

Considering the confusion matrix in Fig. 4, we find the accuracy of classification in RBFNN model for two data sets of training and test. The designed model resulted in 94.8% successful in tree failure classification in the training data set. The accuracy of classification was 90.6% in the test data set and 94% in the all data sets. RBFNN failed 25 samples of 480 samples in the training data set, 11 samples of 120 samples in the test data set, and 36 samples of 600 samples in the all data set.

### Prediction performance of SVM

SVM requires parameter tuning to perform with high accuracy in the best configuration. Indeed, SVM uses some predefined functions, called kernels. The kernel structure drives data into a multi-dimensional space. SVM with Gaussian RBF function combines bell-shaped curves at support vectors. The bell-shaped curves have a particular width which inversely depends on the value of *γ*.

We use the C parameter to the regular simplicity of curves. Indeed, as the value of the C parameter is increased, the classification curve is more intricate. In the RBF function in SVM structure, parameter gamma (γ) is used to modify the system variance and the smoothness of classification boundaries. Table 2 represents prediction accuracies for various SVM parameters of the training and test data.

**Figure 5:**
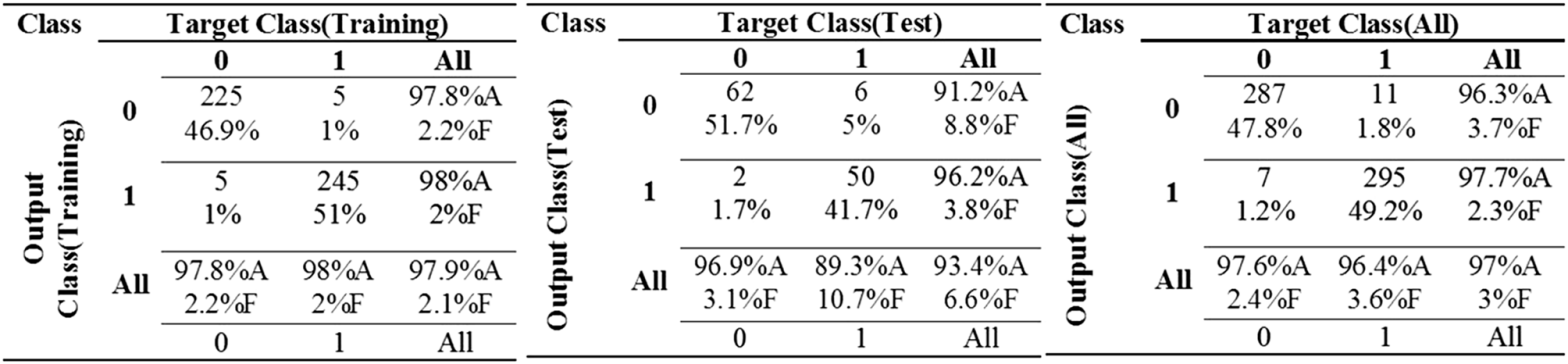
The confusion matrix of SVM neural network.

Considering the accuracy of classification in training and test data sets (Table 2), the value of the most accurate parameter is C = 200 and γ = 24. Considering the confusion matrix in Fig. 5, we find the accuracy of classification in the SVM model for two data sets of training and test. The designed model resulted in 97.9% successful in tree failure classification in the training data set. The accuracy of classification was 93.4% in the test data set and 97% in the all data sets. SVM failed 10 samples of 480 samples in the training data set, eight samples of 120 samples in the test data set, and 18 samples of 600 samples in the all data set.

**Figure 6:**
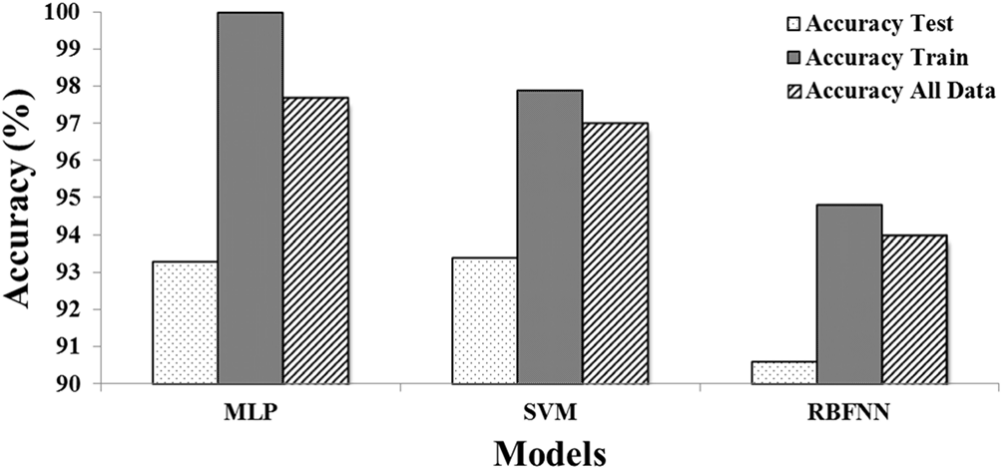
The performance of the designed TFMs for susceptible trees identification.

Considering the results of modeling in Fig. 6, the MLP model is defined as the most accurate model in susceptible tree identification in wind disturbances of forest. Comparing to RBFNN and SVM, the MLP model represents the highest accuracy of target tree classification in data sets. Therefore, TFM_MLP_ (TFM that uses MLP technique) was detected as the most accurate TFM for target tree identification. After randomizing data, we separated training and test data sets so that the same training and test samples were applied for three modeling techniques. Indeed, MLP achieved more accurate results by assigning 20% of all samples for validation during the training process.

### Sensitivity analysis of TFM_mlp_

**Figure 7:**
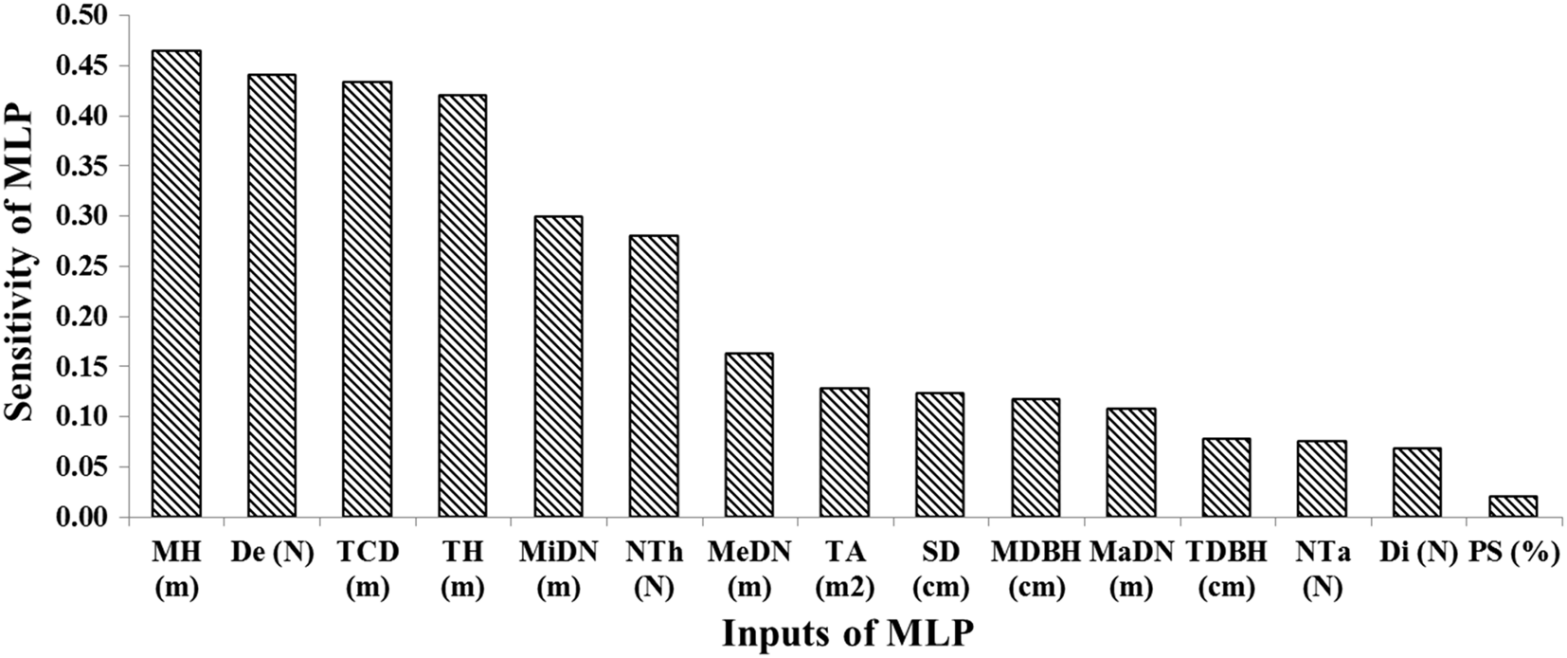
The impact value (0 to 1) of input variables on the tree failure model outputs in sensitivity analysis of model (trees Mean Height (MH), trees Density (De), Tree Crown Diameter (TCD), Tree Height (TH), Minimum Distance from Neighbor trees (MiDN), Number of Thicker trees (than the target tree) (NTh), Mean Distance from Neighbor trees (MeDN), Tree Area (TA), Soil Depth (SD), trees Mean Diameter at the Breast Height (MDBH), Maximum Distance from Neighbor trees (MaDN), Tree Diameter at the Breast Height (TDBH), Number of Taller trees (NTa), trees Diversity (Di) and Plot Slope (PS)).

We performed a sensitivity analysis on the outputs of the optimal TFM_mlp_ model. In Fig. 7, the share of each input variable on model output has been detected by sensitivity analysis. The sensitivity value is the standard deviation of model output changes in response to each variable changes. Considering to the classes of targets, model outputs will be appeared in two classes of 0 and 1. According to the results of sensitivity analysis, the variables of 'the mean of trees height' (0.46) and 'the number of trees (stand density) in plots' (0.44), 'tree crown diameter' (0.43) and 'tree height' (0.42) are prioritized respectively as the most significant inputs which influence tree susceptibility in the windstorm (Fig. 7). Indeed, 'the mean of tree's height' and 'the number of trees (stand density)' are the most influential forest stand factors which increase the chance of tree failure in windstorm events. On the other hand, some tree characteristics increase the vulnerability of trees in windstorms which are 'tree crown diameter' and 'tree height'.

**Figure 8:**
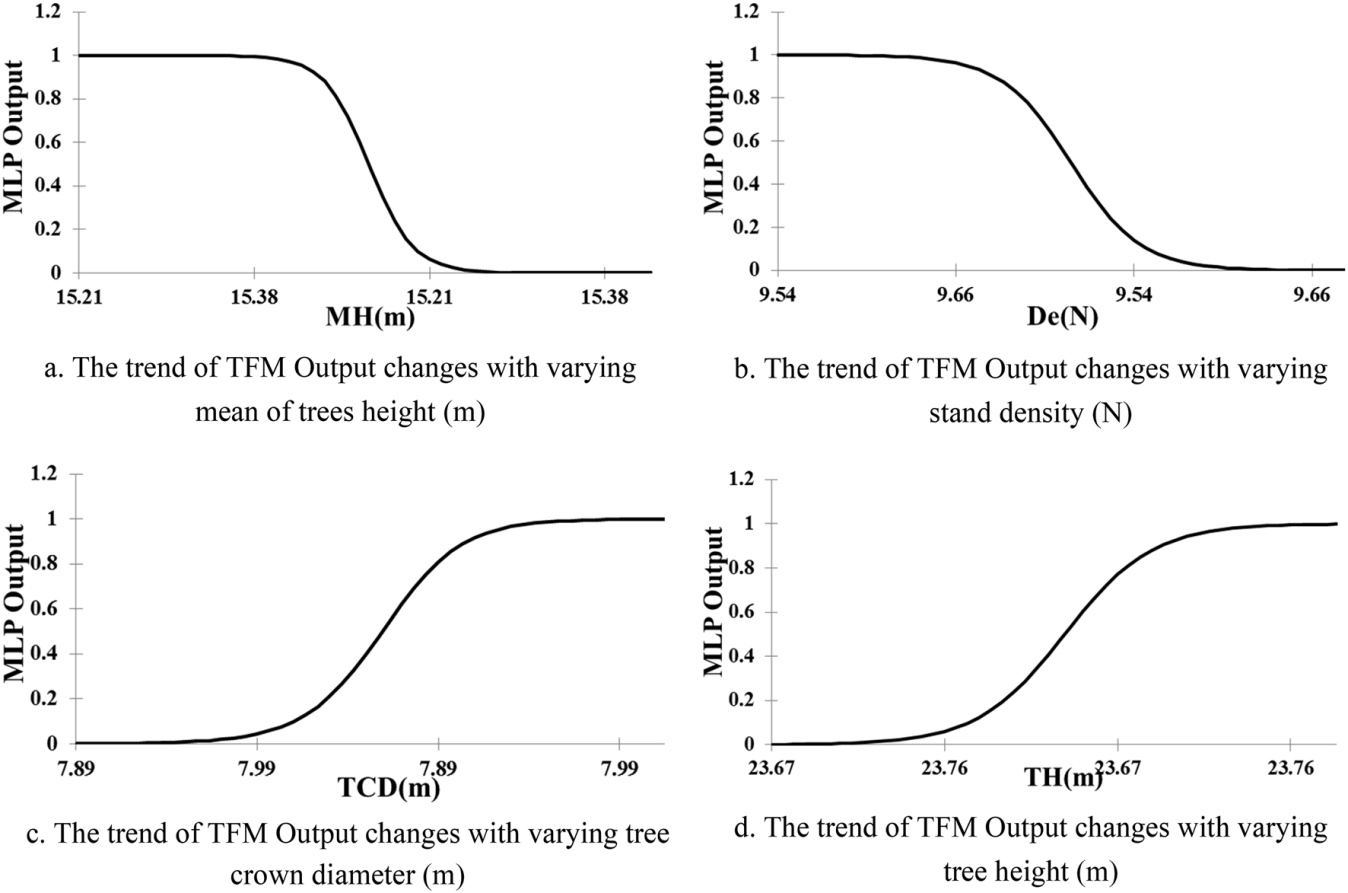
The trend of TFMMLP Output changes with varying the most significant variables.

Considering trends in Fig. 8a,b, 'the mean of stand trees height' and 'stand density' in forest, are negatively correlated to tree susceptibility in the windstorm; so in forest stands where the mean of tree's height and the number of trees (stand density) increase, the probability of trees failure reduces. Indeed, trees in dense forest stands with taller species are less vulnerable in windstorms. Considering trends in Fig. 8a,b, 'tree crown diameter' and 'tree height' in forest, are positively correlated to tree susceptibility in the windstorm so taller trees with high crown dimensions have a remarkable potential to be damaged in the windstorm.

TFM_mlp_ provides a new tool as a decision support system for forest stands management to reduce the number of damaged trees in wind disturbances. The sensitivity analysis prioritizes 'the mean of trees' height', 'the number of trees (stand density) in plots', 'tree crown diameter' and 'tree height' variables as the most significant characteristics of forest trees and stands which influence trees stability in windstorms. These results could be used in forest management and silvicultural methods in respect to the trends.

**Figure 9:**
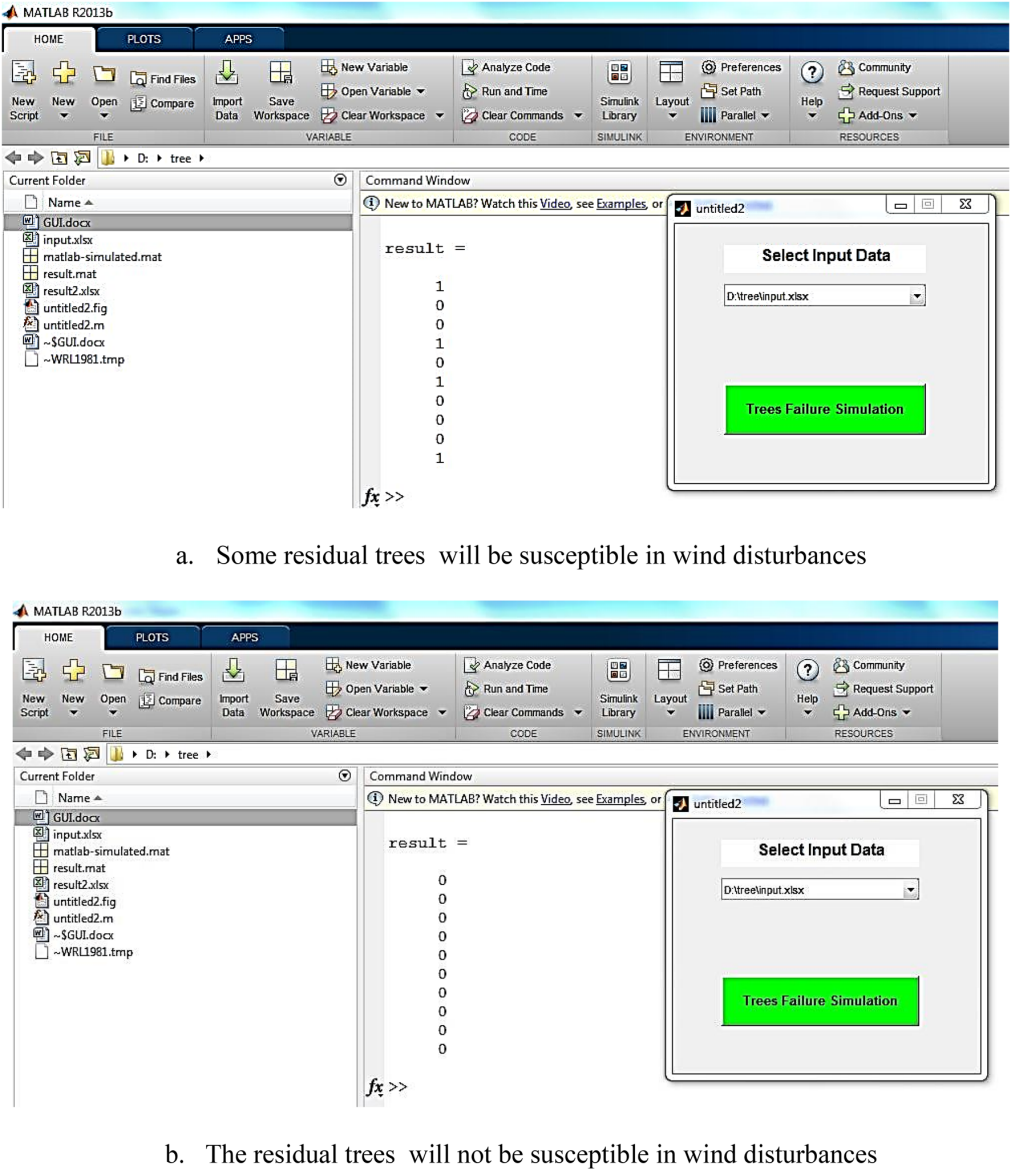
A snapshot of the Graphical User Interface of the Decision Tool for two cases with different plot characteristics (including trees Mean Height (MH), trees Density (De), Tree Crown Diameter (TCD), Tree Height (TH), Minimum Distance from Neighbor trees (MiDN), Number of Thicker trees (than the target tree) (NTh), Mean Distance from Neighbor trees (MeDN), Tree Area (TA), Soil Depth (SD), trees Mean Diameter at the Breast Height (MDBH), Maximum Distance from Neighbor trees (MaDN), Tree Diameter at the Breast Height (TDBH), Number of Taller trees (NTa), trees Diversity (Di) and Plot Slope (PS)).

Finally, a graphical user interface (GUI) was designed to run TFM model on new data when the forest managers are planning for tree cutting. Indeed, after tree harvesting, the characteristics of residual trees and forest stand will be changed. The changes are detectable before harvesting plan implementation by the designed EDSS tool. It means that changes on the tree and stand characteristics are measurable before the implementation of harvesting plan. The forest manager can easily predict the possibility of tree's failure in the windstorm. GUI as an EDSS tool, will be run on new data just by pushing "Tree Failure Simulation" button in Fig. 9. As an example, Fig. 9 illustrates the results of two different tree harvesting plan on ten residual trees. We found four susceptible trees in the plan "a". Therefore, we modified the harvesting plan "a" to plan "b" (changing selected trees for harvesting) and as a result, the trees will be stable in future windstorms. The modification is conducted by changing the most significant factors in sensitivity analysis results and the trends in Fig. 8 (using changes in selected trees for harvesting).

## Discussion

In temperate ecosystems, such as studied Hyrcanian forests, regular and permanent storm damage the environment heavily every year^5,32^. In this research, we have attempted to determine the accuracy of machine learning approaches, namely MLP, RBFNN and SVM in susceptible tree classification. Quantitative models such as regression and ANN were recently developed to model wind throw damage and trees failure^11,33–36^. The most popular empirical approaches use individual tree variables as predictors to develop wind damage models^37,38^. With the aim of models comparison, we developed TFM_MLP_ with 15 trees and stand variables as predictors. In related researches, tree and stand variables have been used for windstorm disturbance modeling, for instance, the model Forest-GALES uses rooting depth and soil type as explanatory variables of regression models that determine tree resistance to be uprooted^39^. The results of our research indicate that the MLP as an ANN modeling approach can very successfully identify wind-susceptible trees during a storm with an accuracy of up to 93.3%. In this paper, we claimed to develop an accurate prediction model to identify the susceptible trees in the windstorm. As the results show, the MLP model identified the susceptible trees in 93.3% of tree samples (120 trees) truly. We believe that this accuracy is acceptable, but future researches can develop more accurate models with considering the other variables of forest stands such as forest edge trees or forest harvesting methods. The reliable results of ANN modeling in tree failure hazard classification have been proved in previous researches, for example Jahani^40^ classified the risk of tree failure in an urban area by SFHR (Sycamore Failure Hazard Risk) model with MLP technique (two classes of tree failure in one and two next years). The results of MLP modeling, especially its remarkable accuracy (97.7%) in comparison with RBFNN (94%), and SVM (97%) results detected TFM_mlp_ as a comparative impact assessment model in susceptible tree identification. It's a rather good idea to have such models to lead into a (sustainable) policy question, and direct legal decisions and questions. Hanewinkel et al.^14^ and Jahani^41^ found that ANN model could identify damaged trees better than the logistic regression models. The authors believe that the prediction of tree failure, in natural conditions of the site, depends on many tree attributes. However, we have limited the studied factors on some plot and tree characteristics. The nominated trees for protection plan would be identified by these factors in studied forest, but the readers should consider the limitation of this research in the application of the model. Indeed, the MLP model would be the first choice in susceptible tree identification modeling under windstorms.

Jahani^11^ designed SFHCM as an environmental decision support system (EDSS) by ANN for tree hazard classification in an urban green space area when the ANN model output is affected by the root damages and the degree of leaning trees. But Hart et al.^19^ indicated that the removal of tree variables such as tree height and tree diameter at breast height in the sensitivity analysis did not have an adverse effect on ANN or regression model performance. On the other hand, they believe that ANN is sensitive to the removal of stand variables (gap size, stand mean diameter at breast height, stand mean height and stand density) and the model performs best when stand variables are available. The results of our research demonstrate that tree and stand variables play the same role in the TFM_mlp_ model outputs so that 'mean of trees height' and 'tree density' variables are negatively, and 'tree crown diameter' and 'tree height' are positively correlated with the susceptibility of trees in the windstorm. However using other factors such as forest edge, land form and tree diversity that are neglected in this research, could be help to improve the accuracy of models.

The developed EDSS tool, such as other proposed EDSS tools for ANN models (Refer to Jahani et al.^17,42^ and Kalantary et al.^52^), is applicable in forest lands where the forest inventory data has been prepared. The new data will be based on forest inventory sample plots and forest managers use these sample plots data to identify susceptible trees for wind-damage. However, forest managers need forest inventory data and maybe the main TFM_mlp_ application would be in protected forest or where the forests are facing windstorms more frequently.

## Conclusions

 Such as other prediction models in forests^43–46,50,51^, TFM_mlp_ was developed for forest managers to assess quickly the impact of cutting trees or thinning stands on tree failure risk in forest. Such as an early warning system^53,54^, TFM_mlp_ would allow simulations of management approaches on the windstorm damage risk. TFM_mlp_ requires extensive data sets of actual wind-damage for its development and could be applied to other forests if the new region conditions are comprehensively covered within TFM_mlp_ training data set. However, TFM_mlp_ could be retrained with new accuracy in any regions that have specific different conditions. The output of model predictions is applied in forest management planning for wood harvesting; especially when the tree cutting plan could be modified based on the designed EDSS tool outputs to reduce the risk of tree's failure in forest wind circulations. The results revealed that MLP was the most accurate technique in susceptible tree identification in windstorm disturbance and the mean of trees' height and density, tree crown diameter and tree's height are respectively the most significant variables which influence tree susceptibility and should be considered in forest activities and tree harvesting.

## Supplementary Information

Supplementary Information.

## Data Availability

The datasets generated during and/or analysed during the current study are available in the Github and GOOGLE DRIVE repository, Data in Github: https://github.com/ajahaniajahani/wind-paper-raw-data. Codes in Github: https://github.com/ajahaniajahani/Wind-paper-data. Data in Google drive: https://docs.google.com/spreadsheets/d/1RorvhLZ_l9j2C-Aw012ypEc3DrwdGtXInVtvnZu83mw/edit?usp=sharing. Codes in Google drive: https://drive.google.com/file/d/1iJak5vqiyOoU6190GVxEJt8knWmQtPMg/view?usp=sharing. Also, all data generated or analysed during this study are included in this published article (and its Supplementary Information files).
